# Validation of digital PCR as a complementary tool to MS2 plaque assay for virucidal activity assessment

**DOI:** 10.3389/fpubh.2026.1850944

**Published:** 2026-07-27

**Authors:** Sung Jae Kim, Song Yi Yang, Min Sik Song, Cheong Ung Kim, Yong Ho Park, Woo Kyung Jung, Jae Myun Lee, Hee Chun Chung

**Affiliations:** 1 Department of Companion Animal Health, Kyungbok University, Namyangju, Republic of Korea; 2 College of Veterinary Medicine and Institute of Veterinary Science, Kangwon National University, Chuncheon, Republic of Korea; 3 Optoraine Co., Ltd., Seongnam, Republic of Korea; 4 Noah Biotech Co., Ltd., Suwon, Republic of Korea; 5 Department of Microbiology and Immunology, Institute for Immunology and Immunological Diseases, Yonsei University College of Medicine, Seoul, Republic of Korea; 6 Department of Microbiology and Immunology, Institute for Immunology and Immunological Diseases, Brain Korea 21 Project for Medical Science, Yonsei University College of Medicine, Seoul, Republic of Korea

**Keywords:** bacteriophage MS2, digital PCR, disinfectant efficacy, double-layer assay, surrogate virus

## Abstract

**Introduction:**

Bacteriophage MS2 is widely applied as a surrogate for pathogenic non-enveloped RNA viruses that are difficult to culture. However, standardized approaches for evaluating disinfectant efficacy using MS2 remain limited. This study aimed to validate dPCR as a rapid quantitative complement to the previously established MS2 plaque assay, assessing their concordance in disinfectant efficacy testing using a previously established double-layer protocol.

**Methods:**

A previously established double-layer MS2 plaque assay was employed. Disinfectant efficacy was tested against two widely used agents−70% ethanol and 60 ppm hypochlorous acid (HOCl)—according to the Korean Disinfectant Efficacy Test Guideline. Viral reduction was determined by plaque counts and further quantified by dPCR following RNase treatment to eliminate free RNA. Agreement between the established plaque assay and dPCR was evaluated using regression and Bland–Altman analyses.

**Results:**

Treatment with 70% ethanol yielded only partial inactivation, with a 2.43-log10 reduction, below the ≥4-log10 benchmark for virucidal efficacy. In contrast, 60 ppm HOCl achieved a 4.38-log10 reduction, confirming effective MS2 inactivation. The dPCR counts correlated strongly with plaque counts (R = 0.999), and Bland–Altman analysis demonstrated excellent agreement within 95% confidence limits, supporting dPCR as a practical alternative to plaque assays. However, the reliability of dPCR as a proxy at low viral concentrations near the limit of detection (<10^2^ PFU/mL) requires further validation.

**Conclusion:**

This study successfully validated dPCR as a rapid quantitative complement to the previously established MS2 double-layer plaque assay, demonstrating its utility in disinfectant efficacy testing. The findings confirm ethanol's limited virucidal capacity against MS2 and HOCl's effectiveness at low concentrations. These results establish dPCR as a promising molecular tool that complements plaque assay results for comprehensive viral surrogate evaluation in disinfection studies.

## Introduction

Effective evaluation of disinfectants is essential for infection control and public health, particularly in the face of emerging viral pathogens (1, 2). Many medically important viruses, including noroviruses, enteroviruses, and highly pathogenic respiratory viruses, are difficult to cultivate or require high biosafety facilities, limiting their direct use in disinfection studies (1–4). To overcome these constraints, surrogate viruses with similar physicochemical and genomic characteristics have been widely employed as safer and more practical alternatives (5). Among these, bacteriophage MS2, a male-specific RNA phage of *Escherichia coli*, has become one of the most widely adopted surrogates for non-enveloped pathogenic viruses (6). MS2 possesses a small icosahedral capsid (25–30 nm) and a single-stranded positive-sense RNA genome of approximately 3.5 kb, resembling key structural and environmental stability features of human enteric viruses such as norovirus and poliovirus (7). Because of its robustness, safety, and ease of propagation, MS2 has been extensively applied in studies of viral persistence, water quality monitoring, and chemical or physical disinfection, including surface, water, aerosol, and UV-based inactivation settings (6, 8–11).

Recent work has also underscored MS2 as a conservative benchmark for disinfectant efficacy compared with other surrogates. Simultaneous testing of bacteriophage MS2 and bovine enterovirus type 1 for foot-and-mouth disease virus disinfectant evaluation demonstrated that MS2 frequently displays higher resistance, supporting its use as a stringent surrogate in regulatory-type studies (8). Despite such widespread use, methodological challenges remain in standardizing MS2-based assays for disinfectant evaluation (8, 12). Conventional plaque assays, while considered the gold standard for infectivity assessment, are labor-intensive, time-consuming, and may show variability due to assay conditions (13). Recent molecular techniques, particularly digital PCR (dPCR), offer highly sensitive and absolute quantification of viral genomes without the need for calibration curves (14), but their concordance with plaque assays in disinfectant efficacy testing requires further validation (15). Moreover, the virucidal performance of commonly used disinfectants against MS2 is not fully established: alcohol-based formulations are widely applied in healthcare and community settings, yet their activity against non-enveloped viruses is often incomplete (16), whereas hypochlorous acid (HOCl) has been reported to exert potent virucidal effects at relatively low concentrations (17, 18).

The primary aim of this study was to validate dPCR as a rapid quantitative complement to a previously established MS2 double-layer plaque assay (12, 19), used here as the gold standard for virucidal testing. Using this assay framework, we compared plaque assay and dPCR readouts during evaluation of two routinely used disinfectants, 70% ethanol and 60 ppm HOCl, to clarify their efficacy against a robust non-enveloped viral surrogate.

## Materials and methods

### Bacteriophage and host bacteria

Bacteriophage MS2 (ATCC 15597-B1) and *Escherichia coli* C3000 (ATCC 15597) were used. MS2 is a non-enveloped, icosahedral RNA phage (25–30 nm, ssRNA ~3.5 kb) specific to F-pili of *E. coli*. Stocks were propagated in C3000 cultures and titrated before experiments (Supplementary Table 1).

### Culture medium

LB broth and agar media (BD Difco, USA) were prepared following the manufacturer's instructions. After autoclaving at 121 C for 15 min, the broth was cooled to 50 C, and a sterile supplement solution was added at 5% (v/v). The supplement consisted of glucose (10 g/L), calcium chloride (0.294 g/L), and thiamine (0.01 g/L) dissolved in 50 mL of deionized water and filtered through a 0.45 μm membrane filter. Glucose and thiamine supported bacterial growth, while Ca^2+^ facilitated successful MS2 DNA insertion into *E. coli* and subsequent phage propagation (12, 20, 21).

For plaque assays, two agar formulations were used: Bottom agar (1.5%): Tryptone (10 g/L), yeast extract (1 g/L), sodium chloride (8 g/L), and agar (15 g/L) dissolved in 950 mL deionized water, autoclaved, cooled to 50 C, and supplemented with 50 mL of the sterile supplement. Approximately 10 mL was dispensed per 90-mm Petri dish and allowed to solidify. Top agar (0.7%): Prepared similarly with 7 g/L agar instead of 15 g/L. After autoclaving and cooling, the supplement was added. The molten top agar was maintained at 45–50 C until use to prevent premature solidification.

This formulation was optimized to ensure clear plaque formation and reproducibility across replicates.

### Bacterial culture solution

*Escherichia coli* C3000 (ATCC 15597) was revived from a freeze-dried stock in 1 mL of supplemented LB broth. The suspension was streaked onto LB agar plates containing supplement and incubated at 37 C for 12–16 h. A single colony was inoculated into 5 mL of supplemented LB broth and cultured at 37 C with shaking for 6 h. The bacterial suspension was adjusted to an optical density of 0.2–0.3 at 600 nm (OD_600_) prior to use in plaque assays, ensuring consistent host cell concentration for phage infection.

### Standard solution of bacteriophage MS2

Freeze-dried bacteriophage MS2 (ATCC 15597-B1) was reconstituted in 1 mL of LB broth. An aliquot of 50 μL (1:100, v/v) was inoculated into the *E. coli* C3000 culture prepared as described above and incubated at 37 C for 16 h with gentle agitation. Following incubation, the culture was centrifuged at 1,800 × g for 10 min to remove bacterial debris. The clarified supernatant was passed through a 0.45 μm membrane filter (Millipore, USA) to obtain a sterile phage suspension. The resulting stock solution was aliquoted and stored at −80 C until further use. Viral titers were determined by plaque assay before experimental applications to ensure consistency across batches.

### Plaque assay (Double-Layer Method)

Serial 10-fold dilutions of MS2 stock (10^−1^ to 10^−12^) were prepared in LB broth. For each dilution, 100 μL of phage suspension was mixed with 10 μL of *E. coli* (OD_600_ = 0.2–0.3). The mixture was combined with molten top agar, gently mixed, and poured onto bottom agar plates. Plates were incubated at 37 C for 16 h. Negative controls consisted of host bacteria without phage, while positive controls used untreated MS2 stock. Plaque numbers were recorded only from plates with 10–100 discrete plaques to ensure reliable quantification. Phage titers were expressed as plaque-forming units per milliliter (PFU/mL). All assays were performed in triplicate, and mean values ± standard deviation were calculated.

### Disinfectant efficacy testing

The virucidal efficacy of 70% ethanol (Wowbiotech, Korea) and 60 ppm hypochlorous acid (HOCl; Soosan CMC, Korea) was evaluated according to the Korean Disinfectant Efficacy Test Guideline (Animal and Plant Quarantine Agency Regulation No. 2022-10). This national standard requires testing under defined organic load conditions and controlled temperature, with a benchmark of ≥4 log_10_ reduction in viral titer to classify a disinfectant as virucidal. Although developed for Korea, the guideline is conceptually consistent with international standards such as EN 14476 (European Norm) and ASTM E1052 (United States), both of which prescribe quantitative suspension tests using viral surrogates, the incorporation of interfering substances to simulate organic load, and demonstration of at least a 4 log_10_ reduction in infectious viral particles under standardized contact times (22). A working suspension was prepared by mixing 1 mL of standardized MS2 stock with 19 mL of organic load solution (hard water containing 5% heat-inactivated fetal bovine serum [FBS; HyClone, Cat. no. SH30919.03, Cytiva, USA]) at 4 C. For each assay, 0.1 mL of the working suspension was added to 4.9 mL of disinfectant solution and incubated at 4 C for 30 min with gentle inversion every 10 min. Control reactions were prepared by mixing 0.1 mL of the working suspension with 4.9 mL of organic load solution and incubated for 30 min under the same conditions as the disinfectant-treated samples. After exposure, 1 mL of each mixture was neutralized with an equal volume of LB broth containing 10% heat-inactivated fetal bovine serum to quench residual disinfectant activity. Residual infectivity was quantified by the double-layer plaque assay, and viral titers were expressed as PFU/mL. Virucidal efficacy was determined by calculating log10 reduction values relative to untreated controls, with a ≥4 log_10_ PFU/mL reduction considered effective.

### Digital PCR application for disinfectant efficacy test

The applicability of digital PCR (dPCR) for disinfectant efficacy testing was evaluated using the neutralized MS2 samples described above. Subsequently, 100 μL of each sample was incubated with 1 μL of RNase A solution (4 mg/mL; Promega, USA) at 37 C for 1 h, followed by inactivation using 2 μL of RNase inhibitor (20 U/μL; Invitrogen, USA). This step was intended to reduce the background signal from free viral genetic RNA. Viral RNA was then extracted using the QIAamp Viral RNA Mini Kit (Qiagen, USA). RNA purity and integrity were confirmed by spectrophotometry (A260/A280 ≥ 1.8) and verified in selected samples using a NanoPhotometer^®^ (Implen, N60/N50). dPCR reactions were performed on the LOAA dPCR platform (Optolane, Korea) with MS2-specific primers and a hydrolysis probe targeting the coat protein gene (Supplementary Table 2). Each 30 μL reaction contained 5 μL of RNA template, 15 μL of 2 × qRT-premix (Biofact, Korea), and 1 μL of each primer and probe (10 μM each for primers and 5 μM for the probe). Cycling conditions consisted of reverse transcription at 50 °C for 10 min, initial denaturation at 95 °C for 10 min, and 40 amplification cycles (95 °C for 15 s, 60 °C for 30 s).

All reactions included no-template controls (NTCs) and positive controls using purified MS2 RNA to confirm assay specificity. Each sample was analyzed in triplicate. Results were expressed as copies/μL, log10-transformed, and compared with plaque assay counts. Agreement between the two methods was assessed using linear regression and Bland–Altman analysis, and statistical significance was determined at p < 0.05.

## Results and discussion

The previously established MS2 double-layer plaque assay (12, 19) with slight modification produced reproducible plaques (Supplementary Figure 1), serving as the validated reference standard for dPCR comparison. In this study, the plaque assay was successfully performed with high consistency, providing a reliable baseline for evaluating the accuracy and performance of the dPCR method.

### Comparative efficacy of ethanol and HOCl

Ethanol is among the most widely used disinfectants, acting rapidly against a broad spectrum of microorganisms (23). Its virucidal activity is highly effective against enveloped viruses, targeting the lipid membrane, but it is less consistent against non-enveloped viruses (24). In this study, 70% ethanol reduced MS2 infectivity by only 2.43 log_10_ PFU/mL (Table 1), which falls short of the ≥4 log10 benchmark required to establish virucidal activity. This experimental outcome mirrors observations in noroviruses, structurally similar to MS2, where capsid resilience reduces alcohol sensitivity (25).

In contrast, hypochlorous acid (HOCl) demonstrated strong virucidal efficacy. Even at a relatively low concentration (60 ppm), HOCl reduced MS2 infectivity by 4.38 log10 PFU/mL (Table 1), surpassing the efficacy threshold. HOCl acts by oxidizing thiol-containing amino acids, inducing protein unfolding, and disrupting viral surface structures (18, 26, 27). These findings highlight a practical implication: while alcohol-based formulations may be insufficient against non-enveloped viruses in healthcare and food environments, HOCl represents a cost-effective and environmentally sustainable option, consistent with prior reports of its broad-spectrum activity at 20–200 ppm (28–30).

**Table 1:** Disinfectant efficacy of 70% ethanol and 60 ppm HOCl against bacteriophage MS2, expressed as PFU/mL and log_10_ reductions determined by plaque assay.

Experiment	Sample	Plaque count (PFU/ml)	Average (Log_10_ transformation)
Double layer assay	Control	1.0 × 10^8^	6.5 × 10^7^ PFU/ml (7.81)
		3.5 × 10^7^	
		5.8 × 10^7^	
	70% ethanol treated	2.8 × 10^5^	2.4 × 10^5^ PFU/ml (5.38)
		2.3 × 10^5^	
		2.0 × 10^5^	
	60 ppm HOCl treated	2.4 × 10^3^	2.7 × 10^3^ PFU/ml (3.43)
		1.2 × 10^3^	
		4.7 × 10^3^	

### Digital PCR as a complementary quantification method

**Figure 1:**
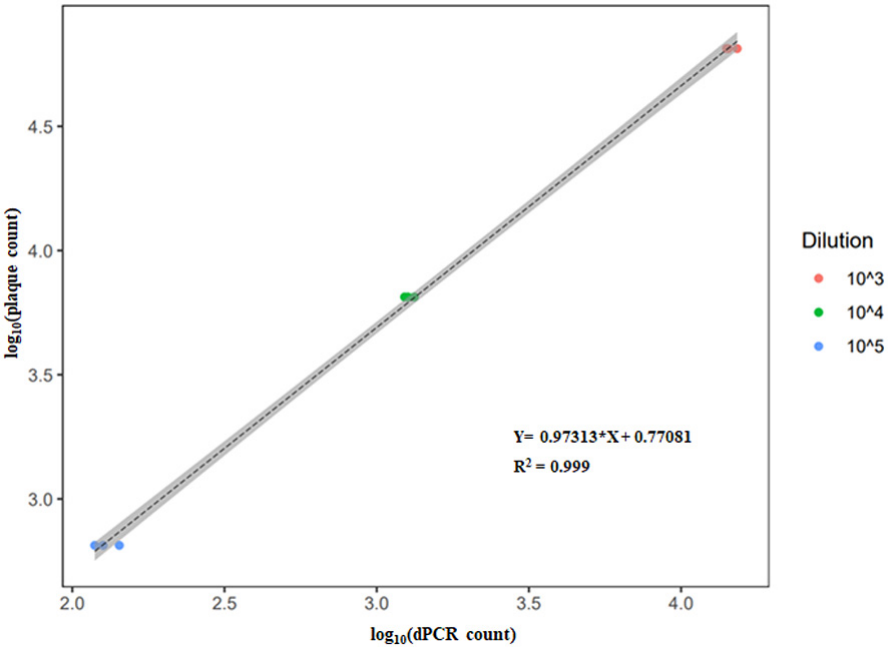
Regression analysis of bacteriophage MS2 quantification by digital PCR (dPCR) compared with plaque assay. Log10-transformed copy numbers obtained by dPCR were plotted against plaque-forming unit (PFU) counts from the double-layer assay using control samples. A highly linear correlation was observed (R = 0.999), indicating strong agreement between the two methods. The regression equation is shown, and the shaded area around the line represents the 95% confidence interval. This result supports the reliability of dPCR as an alternative method for disinfectant efficacy testing.

**Figure 2:**
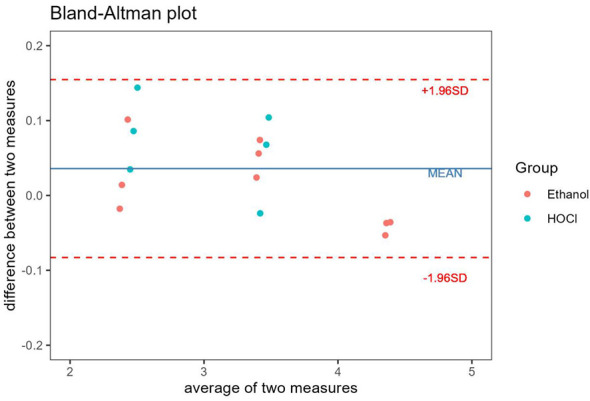
Bland–Altman analysis comparing plaque assay results with PFU values inferred from the dPCR regression equation. The horizontal axis represents the mean of the two methods (dPCR-inferred PFU and actual PFU), while the vertical axis represents the difference between them (inferred PFU minus actual PFU). The solid line indicates the mean of the differences (0.036), and the dashed lines denote the 95% limits of agreement [calculated as the mean of the differences ± 1.96 × standard deviation (SD); range: −0.083 to 0.155]. Each data point corresponds to an individual paired measurement (*n* = 15) across various dilutions of disinfectant-treated groups (ethanol-treated in red; HOCl-treated in blue). All 15 paired measurements lie within the 95% limits of agreement, indicating strong concordance between dPCR and plaque assay results across all conditions.

Traditional plaque assays, while considered the gold standard for infectivity, are labor-intensive and time-consuming. Digital PCR (dPCR) offers absolute quantification of viral genomes without reliance on standard curves, unlike qPCR, which is semi-quantitative (31, 32). In this study, we validated dPCR against plaque assay results for MS2. To establish a correlation between dPCR and the plaque assay, control samples were diluted across three dilution levels (10^3^–10^5^). The log_10_-transformed dPCR values and log-transformed PFU values from these samples were analyzed using linear regression to derive the regression equation. This equation (log_10_ [PFU/mL] = 0.973 × log_10_ [dPCR copies/μL] + 0.771; *R* = 0.999) demonstrated excellent concordance (Figure 1). For each disinfectant-treated sample, appropriate dilution factors (10^0^–10^3^) were selected based on the initial viral concentration. After performing dPCR on these diluted samples, the log_10_-transformed dPCR values were converted into estimated log_10_-transformed PFU values using the regression equation (Table 2). Subsequently, the estimated PFU values derived from the regression equation were compared with the actual PFU values obtained from the plaque assay using Bland-Altman analysis (33). The Bland-Altman analysis confirmed minimal bias, with all values falling within the 95% limits of agreement (Figure 2). This concordance validates dPCR as a reliable molecular complement to infectivity-based assays.

**Table 2:** Digital PCR quantification of MS2 after disinfectant treatment, with results compared to plaque assay values using regression analysis.

Experiment		Digital PCR			Double layer assay	
Sample	Dilution	Copies/ul	Log_10_- transformation	Plaque count (log_10_-transformed) inferred by regression equation	Plaque count (PFU/ml)	Log_10_- transformation
Control	10^3^	14,289.23	4.16	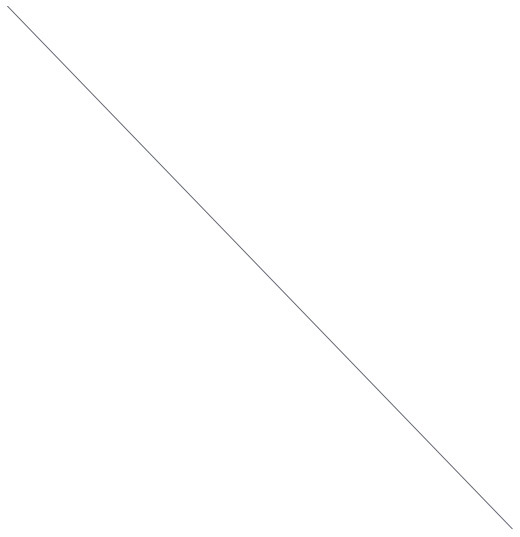	65,000	4.81
		15,282.47	4.18			
		14,106.75	4.15			
	10^4^	1,328.21	3.12		6,500	3.81
		1,265.34	3.10			
		1,235.13	3.09			
	10^5^	126.21	2.10		650	2.81
		142.72	2.15			
		118.48	2.07			
70% ethanol treated	10^1^	4,702.41	3.67	4.34	24,000	4.38
		4,689.92	3.67	4.34		
		4,512.26	3.65	4.33	
	10^2^	572.25	2.76	3.45	2,400	3.38
		508.23	2.71	3.40		
		548.34	2.74	3.44	
	10^3^	43.21	1.64	2.36	240	2.38
		46.59	1.67	2.39		
		57.28	1.76	2.48	
60 ppm HOCl treated	10^0^	693.51	2.84	3.54	2,700	3.43
		636.27	2.80	3.50		
		512.24	2.71	3.41	
	10^1^	71.51	1.85	2.58	270	2.43
		55.23	1.74	2.47		
		62.34	1.79	2.52	

Beyond simple correlation, dPCR offered clear advantages in reproducibility and sensitivity. Its partitioning system allowed reliable detection of low-copy viral genomes, a critical feature for monitoring residual genetic targets after chemical inactivation (34). By eliminating dependence on calibration curves, dPCR inherently reduces inter-laboratory variability and improves the comparability of quantitative data (35). Furthermore, from a practical standpoint, dPCR provides results within the same day (~5–6 h), whereas plaque assays require overnight incubation (~16 h), resulting in a shorter turnaround time.

In the specific context of virucidal efficacy testing, the proposed dPCR platform offers critical methodological merits over both conventional qPCR and the gold-standard plaque assay. First, a major challenge in evaluating disinfectants (e.g., ethanol, HOCl) via molecular methods is the carryover of chemical residuals, which frequently act as potent PCR inhibitors and lead to false-negative or underestimated results in standard qPCR (15, 36). Because dPCR partitions the reaction mixture into thousands of independent nanoliter droplets, the effective concentration of these inhibitors within any single positive partition is drastically minimized. This unique partitioning technology grants dPCR a significantly higher tolerance to complex chemical matrices, ensuring robust and reliable quantification without the need for extensive sample purification that might otherwise lead to the loss of viral nucleic acids.

Second, to address the inherent challenge in molecular quantification where naked or residual viral nucleic acids from inactivated particles can lead to overestimation, our workflow integrates a RNase A pretreatment prior to dPCR amplification. Since RNase A specifically cleaves exposed single-stranded RNA at the 3′ side of pyrimidine residues, this step was designed to reduce the background signal from free genetic debris. Consequently, the remaining dPCR signal does not merely count total viral copies; rather, it quantifies encapsulated, structurally intact genomes, thereby serving as a promising molecular proxy for actual viral infectivity.

These findings demonstrate excellent concordance between dPCR and the established MS2 plaque assay (R = 0.999, Figure 1; Bland-Altman within 95% limits of agreement, Figure 2), validating this dPCR approach as a rapid, quantitative complement for virucidal testing. From a practical standpoint, the combined use of plaque assays and dPCR provides a balanced, synergistic framework: while traditional plaque assays ensure biological relevance, the dPCR platform delivers the speed, absolute precision, and high-throughput scalability required for screening novel virucidal agents. This integrated strategy enhances the reliability of disinfectant validation and supports regulatory assessments, particularly for non-enveloped viruses where current testing protocols are insufficient. However, while a robust linear correlation was confirmed within the tested range, the exact limit of detection (LOD) and the performance of this relationship below 10^2^ PFU/mL has not yet been fully established. This limitation is particularly important when assessing residual infectivity following disinfection, where viral titers may approach the detection limit. Given that monitoring residual infectivity at low viral titers is critical for rigorous virucidal assessment, further studies are warranted to validate the concordance of dPCR and plaque assay results in low titer ranges.

Because dPCR retains sensitivity in complex matrices, the approach can extend beyond laboratory conditions to applications in water safety monitoring, food hygiene surveillance, and hospital decontamination (34, 36). Importantly, these findings contribute to international standardization efforts by providing reproducible surrogate-based assays that align with both national and global guidelines (19, 22). In doing so, the study not only advances methodological rigor but also strengthens preparedness for emerging viral threats through harmonized, evidence-based disinfection protocols.

### Limitations and future directions

This study has several limitations that warrant consideration. First, while MS2 serves as a practical surrogate for non-enveloped human viruses, structural and biological differences from clinically relevant pathogens may limit direct extrapolation of results (2, 37). Second, digital PCR detects viral genomes regardless of infectivity, and thus residual RNA from inactivated particles may lead to overestimation in certain contexts (15, 38, 39). Third, as a preliminary validation study, the sample size and number of replicates were limited. While promising concordance was observed (R = 0.999), larger-scale studies with expanded sample sizes and more extensive replicates are needed to confirm that dPCR achieves high consistency with actual plaque assay results and can serve as a practical substitute. Future research should conduct comprehensive validation with larger sample sizes and multiple replicates to definitively establish dPCR as a robust alternative to plaque assay. Additional phage surrogates and different disinfectants should be tested to broaden applicability. Multi-laboratory studies across different phage-host systems would strengthen generalizability and standardization for regulatory virucidal testing.

## Conclusion

In conclusion, this study successfully validated digital PCR (dPCR) as a high-precision, rapid alternative to the traditional plaque assay for evaluating the virucidal efficacy of disinfectants against non-enveloped viruses. Using bacteriophage MS2 as a surrogate, we demonstrated that while 70% ethanol was insufficient to achieve the required 4-log10 reduction, hypochlorous acid (HOCl) at 60 ppm provided potent virucidal activity, surpassing regulatory thresholds.

The most significant finding of this research is the exceptional concordance (R = 0.999) between dPCR and the gold-standard plaque assay. By incorporating an RNase treatment step following disinfection, we effectively eliminated free viral RNA from damaged particles, allowing dPCR to selectively quantify encapsidated, potentially infectious viral genomes. This methodological refinement addresses the long-standing limitation of molecular assays—the inability to distinguish between infectious and inactivated viruses—thereby positioning dPCR as a promising “molecular infectivity assay.”

The Bland–Altman analysis further confirmed the minimal bias and high reproducibility of dPCR, which offers distinct advantages in terms of speed, sensitivity, and scalability compared to labor-intensive culture methods. While the current study utilized a limited number of replicates, the near-perfect correlation justifies the transition toward dPCR-based frameworks for faster disinfectant validation.

Ultimately, the integration of dPCR into virucidal testing protocols enhances methodological rigor and supports international standardization efforts. This approach provides a robust, evidence-based tool for public health preparedness, ensuring rapid and accurate assessment of disinfection strategies against emerging viral threats in clinical, food, and environmental settings. Further studies are required to validate the reliability of dPCR as a proxy for infectivity at low viral concentrations near the limit of detection, where accurate assessment of residual infectivity is critical.

## Data Availability

The original contributions presented in the study are included in the article/Supplementary material, further inquiries can be directed to the corresponding authors.
