# Supplementary material for: Validation of digital PCR as a complementary tool to MS2 plaque assay for virucidal activity assessment
Source: Front Public Health. 2026 Jul 27;14:1850944. doi: 10.3389/fpubh.2026.1850944 (PMC13454040; doi:10.3389/fpubh.2026.1850944)
Supplement: Supplementary file 1 [file Data_Sheet_1.docx]

Supplementary Table 1. Characteristics of bacteriophage MS2

| Host | *Escherichia coli* C3000 (ATCC 15597) |
| --- | --- |
| Family/Genus | *Leviviridae/Levivirus* |
| Phage type | group I male-specific RNA phage |
| Genome | ssRNA (+), length 3.5 ~ 3.6 kb |
| Morphology | Non-eveloped, icosachedral (25~30 nm in diameter) |

Supplementary Table 2. Primer set for detecting bacteriophage MS2

| Primer | 5’ – 3’ oligo sequences | References |
| --- | --- | --- |
| MS2-F | GGGAGTACAATCCGTATGGC | In this study  (GenBank.M25187) |
| MS-R | AGAGAGCCGTTGCCTGATTA |  |
| MS2-Probe | ACTGGCGCGTACGTAAAGTCTCCT |  |

Supplementary Figure 1. MS2 double-layer plaque assay.

(A) Plaque formation across serial 10-fold dilutions of bacteriophage MS2; black handwritten symbols on each plate indicate the corresponding dilution factors used for titer determination. (B) Control plate containing only the host bacterium *Escherichia coli* C3000 without MS2 infection. (C) Negative control plate in which 100 µL of phosphate-buffered saline (PBS) was inoculated into the top agar instead of phage-containing suspension.
